# Supplementary material for: Comparative genomics provides new insights into the diversity, physiology, and sexuality of the only industrially exploited tremellomycete: Phaffia rhodozyma
Source: BMC Genomics. 2016 Nov 9;17:901. doi: 10.1186/s12864-016-3244-7 (PMC5103461; doi:10.1186/s12864-016-3244-7)
Supplement: Additional file 6: — List of orphan genes with links to PFAM (related to Additional file 1: Table S1). (ZIP 1428 kb) [file 12864_2016_3244_MOESM6_ESM.zip › BLAST_HTML_FTR/G05237_P.html]

BLAST Search Results


```
BLASTP 2.2.27+


Reference:
Stephen F. Altschul, Thomas L. Madden, Alejandro A. Schäffer,
Jinghui Zhang, Zheng Zhang, Webb Miller, and David J. Lipman (1997),
"Gapped BLAST and PSI-BLAST: a new generation of protein database
search programs", Nucleic Acids Res. 25:3389-3402.


Reference for
composition-based statistics:
Alejandro A. Schäffer, L. Aravind, Thomas L. Madden, Sergei
Shavirin, John L. Spouge, Yuri I. Wolf, Eugene V. Koonin, and
Stephen F. Altschul (2001), "Improving the accuracy of PSI-BLAST
protein database searches with composition-based statistics and
other refinements", Nucleic Acids Res. 29:2994-3005.


Database: nr
           71,551,133 sequences; 26,053,659,533 total letters


Query= G05237_P

Length=491
                                                                      Score     E
Sequences producing significant alignments:                          (Bits)  Value

emb|CDZ97572.1|  hypothetical protein [Xanthophyllomyces dendrorh...   559    0.0  
gb|KKA70638.1|  isl-1 [Pristionchus pacificus]                        39.7    2.9  
gb|KMQ94316.1|  hypothetical protein RF55_5543 [Lasius niger]         39.7    9.5  


 >emb|CDZ97572.1| hypothetical protein [Xanthophyllomyces dendrorhous]
Length=394

 Score =  559 bits (1440),  Expect = 0.0, Method: Compositional matrix adjust.
 Identities = 288/312 (92%), Positives = 288/312 (92%), Gaps = 23/312 (7%)

Query  188  MVNVDSEETQSENVCQFCIEPRSIVTAQEHDQAVFHHLEPGVSSKTTAVDPIWAPGKDGS  247
            MVNVDSEETQSENVCQFCIEPRSIVTAQEHDQAVFHHLEPGVSSKTTAVDPIWAPGKDGS
Sbjct  1    MVNVDSEETQSENVCQFCIEPRSIVTAQEHDQAVFHHLEPGVSSKTTAVDPIWAPGKDGS  60

Query  248  DDSEGPFDPNIICCPLPIDELVPSSPLLCSSPMVEIDMPELWSLDGETIEEEEEKEKEPD  307
            DDSEGPFDPNIICCPLPIDELVPSSPLLCSSPMVEIDMPELWSLDGETIEEEEEKE EPD
Sbjct  61   DDSEGPFDPNIICCPLPIDELVPSSPLLCSSPMVEIDMPELWSLDGETIEEEEEKETEPD  120

Query  308  VIVESGDSKTGVWTIDGVQSIQDHS-----------------------DTLNWYGHLYLQ  344
            VIVESGDSKTGVWTIDGVQSIQDHS                       DTLNWYGHLYLQ
Sbjct  121  VIVESGDSKTGVWTIDGVQSIQDHSLTSTLRPFNTTTLPGGLLSLIKHDTLNWYGHLYLQ  180

Query  345  HILSTAVNIFAPSPAAFRSKTGFELTIYLREVANLPLIGMGPSVSAEFLSLSAQADMQMK  404
            HILSTAVNIFAPSPAAFRSKTGFELTIYLREVANLPLIGMGPSVSAEFLSLSAQADMQMK
Sbjct  181  HILSTAVNIFAPSPAAFRSKTGFELTIYLREVANLPLIGMGPSVSAEFLSLSAQADMQMK  240

Query  405  EEKEDTPSSKNLDSSTEISDEATPVQEPMRGQLHGNLCKKCSWYMGCSSEKGSTSEYSSK  464
            EEKEDTPSSKNLDSSTEISDEATPVQEPMRGQLHGNLCKKCSWYMGCSSEKGSTSEYSSK
Sbjct  241  EEKEDTPSSKNLDSSTEISDEATPVQEPMRGQLHGNLCKKCSWYMGCSSEKGSTSEYSSK  300

Query  465  TLVMTSVELSAQ  476
            TLVMTSVELSAQ
Sbjct  301  TLVMTSVELSAQ  312


>gb|KKA70638.1| isl-1 [Pristionchus pacificus]
Length=170

 Score = 39.7 bits (91),  Expect = 2.9, Method: Compositional matrix adjust.
 Identities = 22/64 (34%), Positives = 40/64 (63%), Gaps = 2/64 (3%)

Query  126  PFRWTR-HSKTSSSSSSSSTSSSVDIN-RKPANGGLKIKLKFKRMKGHGMHAIYELFGGS  183
            P+R+ R +++ SS   + +T +  D + R+P++G LK+K +  RMK   +H++Y L G S
Sbjct  89   PYRFLRPYARVSSVGHNLATYTDADRDFRRPSDGRLKMKNRAVRMKNPSLHSLYSLTGES  148

Query  184  KIRT  187
            + R 
Sbjct  149  EERA  152


>gb|KMQ94316.1| hypothetical protein RF55_5543 [Lasius niger]
Length=1387

 Score = 39.7 bits (91),  Expect = 9.5, Method: Compositional matrix adjust.
 Identities = 53/240 (22%), Positives = 96/240 (40%), Gaps = 36/240 (15%)

Query  2    FHHSRPKDPQQEVHRSSLSRVLHSDLYLARHRPCPTRTSTAPEFLSAPLSSLRGGDTTLA  61
            F  +R K P+ E   S +++VL  D+ +   +   TR++  PE       S++  +T+  
Sbjct  646  FDCNRDKVPEDEAVSSRVAKVL--DILVEAEKDGKTRSAVQPE------RSIQDKETSRL  697

Query  62   ETTSVCEYDIGQIETVILNSDDTPITSDYAQHQHHHQRHPPQHHPHSQPHPNRPEI----  117
               S  +   G+ ++ +LN+ + PIT+D  +     +   P+     +P  N  ++    
Sbjct  698  -INSNQKAKSGKTKSTVLNTVEDPITADLKEMLKEMKHSLPKRPKPRKPMSNDGDVLEKV  756

Query  118  -------SRRATLAHPFRWTRHSKTSSS-------SSSSSTSSSVDINR---------KP  154
                   + + +       TR   TSS+         SSS  +S +I R         +P
Sbjct  757  DKRSPVSANKTSYVASITTTRMEATSSNDYEADKQKVSSSAQTSGNIRRLNQPSTSAERP  816

Query  155  ANGGLKIKLKFKRMKGHGMHAIYELFGGSKIRTMVNVDSEETQSENVCQFCIEPRSIVTA  214
            +  G K +    R  G G   +   F   + R    +++ +T  EN     IEP   V A
Sbjct  817  STSGWKHENVLYRTSGKGSALVKNTFQLIRPRDFAEIEATKTTKENAYANMIEPSLYVNA  876


Lambda      K        H        a         alpha
   0.313    0.128    0.381    0.792     4.96 

Gapped
Lambda      K        H        a         alpha    sigma
   0.267   0.0410    0.140     1.90     42.6     43.6 

Effective search space used: 5027646596448


  Database: nr
    Posted date:  Sep 23, 2015 12:05 AM
  Number of letters in database: 26,053,659,533
  Number of sequences in database:  71,551,133


Matrix: BLOSUM62
Gap Penalties: Existence: 11, Extension: 1
Neighboring words threshold: 11
Window for multiple hits: 40
```
